# An Exploration of Non-Coding RNAs in Extracellular Vesicles Delivered by Swine Anterior Pituitary

**DOI:** 10.3389/fgene.2021.772753

**Published:** 2021-11-29

**Authors:** Jiali Xiong, Haojie Zhang, Bin Zeng, Jie Liu, Junyi Luo, Ting Chen, Jiajie Sun, Qianyun Xi, Yongliang Zhang

**Affiliations:** ^1^ Guangdong Provincial Key Lab of Agro-Animal Genomics and Molecular Breeding, College of Animal Science, National Engineering Research Center for Breeding Swine Industry, South China Agricultural University, Guangzhou, China; ^2^ College of Animal Science and Technology, Guangxi University, Nanning, China

**Keywords:** anterior pituitary extracellular vesicles, miRNA, lncRNA, circRNA, cross-talk

## Abstract

Extracellular vesicles are lipid bilayer-delimited particles carrying proteins, lipids, and small RNAs. Previous studies have demonstrated that they had regulatory functions both physiologically and pathologically. However, information remains inadequate on extracellular vesicles from the anterior pituitary, a key endocrine organ in animals and humans. In this study, we separated and identified extracellular vesicles from the anterior pituitary of the Duroc swine model. Total RNA was extracted and RNA-seq was performed, followed by a comprehensive analysis of miRNAs, lncRNAs, and circRNAs. Resultantly, we obtained 416 miRNAs, 16,232 lncRNAs, and 495 circRNAs. Furthermore, GO and KEGG enrichment analysis showed that the ncRNAs in extracellular vesicles may participate in regulating intracellular signal transduction, cellular component organization or biogenesis, small molecule binding, and transferase activity. The cross-talk between them also suggested that they may play an important role in the signaling process and biological regulation. This is the first report of ncRNA data in the anterior pituitary extracellular vesicles from the duroc swine breed, which is a fundamental resource for exploring detailed functions of extracellular vesicles from the anterior pituitary.

## Introduction

The pituitary gland is often regarded as the “master gland”, coordinating the complex functions of multiple endocrine glands along with the hypothalamus (Barkhoudarian 2017). The anterior glandular lobe of the pituitary, namely, the anterior pituitary, is a very important organ of the endocrine system that regulates several physiological processes including cell generation cycle, stress response, growth, reproduction, bone metabolism, and lactation (Schally et al., 1977; Weiss et al., 1978; Lin et al., 1983; Rocha et al., 2003; Takeuchi, 2009). It accounts for 80% of the entire pituitary gland and secretes six major hormones, including growth hormone (GH), prolactin (PRL), adrenocorticotropin hormone (ACTH), thyroid-stimulating hormone (TSH), luteinizing hormone (LH), and follicle-stimulating hormone (FSH), which are crucial to our physiological wellbeing (Nelson 2005; Le Tissier et al., 2012). These hormones target the adrenal gland, liver, bone, thyroid, breast, ovary, and testes, which are themselves regulated by the negative feedback of the hypothalamus and these target organs (Schally et al., 1977; Lin et al., 1983; Barkhoudarian, 2017).

Extracellular vesicles (EVs) are a type of nano-scale vesicles that can be secreted by most eukaryotic cells (van Niel et al., 2018; Jiang et al., 2021). EVs usually have cup- or round-shaped phospholipid bilayers under transmission electron microscopy, and are mainly spherical in body fluids. They are present in various tissues and biological fluids including blood, dendritic cells, lymphocytes, epithelial cells, red blood cells, stem cells, hepatocytes, and various tumor cells (Raposo et al., 1996; Zitvogel et al., 1998; Wolfers et al., 2001; Blanchard et al., 2002; Keller et al., 2006; Cabili et al., 2011; Regev-Rudzki et al., 2013; Han et al., 2016; Ibrahim et al., 2016), carrying a cargo of biological molecules of their origin, including proteins, lipids, mRNAs, microRNAs (miRNAs), long non-coding RNAs (lncRNAs), and circular RNAs (circRNAs) (Zitvogel et al., 1998; Thery et al., 2002; Keller et al., 2006; Koppers-Lalic et al., 2014). Latest data from Exocarta database show that 9,769 proteins, 3,408 mRNAs, and 2,838 miRNAs have been identified in EVs of different cellular origin (http://www.Exocarta.org). EVs were previously considered to be a waste of protein produced during cell metabolism (Johnston, 1992) until researchers found in the 1990s that they have immunoregulatory functions and can be an important cell regulatory factor (Raposo et al., 1996). More and more evidence showed that EVs have multiple functions in intercellular communication, which can be involved in the material transfer, signal transduction, and immune response regulation (Natasha et al., 2014; Pan et al., 2017; Raghu, 2016; Gang et al., 2018). Recently, Zhang et al. reported that pituitary tumor EVs inhibit the growth of pituitary adenoma by transmitting lncRNA H19 (Zhang et al., 2019).

Non-coding RNA (ncRNA) is a variety of functional RNA molecules that would not be translated into proteins. MiRNA is a type of small ncRNA and can negatively regulate the expression of its target gene expression at the post-transcriptional level (Bartel, 2018; Bartel, 2004; Doench et al., 2004). MiRNAs can participate in regulating the development of the pituitary gland (Calin et al., 2002; Bottoni et al., 2005; Amaral et al., 2009; Mao et al., 2010; Zhang et al., 2010; Nemoto et al., 2012; Schneeberger et al., 2012; Choi et al., 2013; Nemoto et al., 2013; Ye et al., 2013; Zhang et al., 2013a; Lannes et al., 2015). LncRNA is another type of ncRNA, defined as transcripts with longer than 200 nucleotides (Esteller, 2011). Researches indicated that lncRNAs play an important part in various biological processes (Li et al., 2015a; Mattick and Rinn 2015) and function in pituitary adenomas and normal anterior pituitary (Chunharojrith et al., 2015; Li et al., 2015b; Han et al., 2017; Fu et al., 2018). CircRNA is a class of single-stranded RNA that forms a covalently closed continuous loop. They were categorized as ncRNA, but more recently, they have been shown to code for proteins and could serve as miRNA sponges and compete with miRNAs to bind target mRNAs (Hansen et al., 2013; Memczak et al., 2013; Pamudurti et al., 2017). Many studies characterize circular RNAs by sorting through vast collections of RNA sequencing data (Salzman et al., 2012; Jeck et al., 2013; Memczak et al., 2013; Boeckel et al., 2015). Recently, Li et al. identified 6,113 circRNAs from the muscle of prenatal and postnatal sheep through RNA-seq (Li et al., 2017b), and some other studies have reported about circRNAs in pituitary adenomas (Liu et al., 2009; Wang et al., 2018).

As an important endocrine organ, there were very limited information about the secretion of EVs in the anterior pituitary gland. In this study, we extracted and identified EVs from the anterior pituitary of Duroc swine breed for the first time, and we also explored its ncRNAs. This study will provide a basis for further exploration of the functions of pituitary EVs.

## Materials and Methods

### Sample Collection and EV Isolation

This study used three healthy male swine (Duroc) at 60 days of age, which were purchased from the Jintuan farm of JIADA GROUP (Zhaoqing, Guangdong, China). An endotracheal tube (30 cm length, 8 mm ID) was used to anesthetize the pigs with isoflurane (4.5% of tidal volume by mask) (Jantzen et al., 2011). Then, the pigs were euthanized by exsanguination under a surgical plane of the isoflurane anesthesia (Laber et al., 2016). The pituitary glands were removed, and the anterior lobe was immediately dissected under sterile conditions, rinsed in phosphate-buffered saline (PBS), and transferred to Hanks’ balanced salt solution. The anterior pituitary tissue was cut up into 1 mm^3^ pieces and cultured in serum-free Dulbecco’s modified Eagle’s medium/nutrient mixture F12 (DMEM/F12) (Gibco, US) supplemented with 100 U/ml penicillin and 100 μg/ml streptomycin (Gibco, US). Forty-eight hours later, the conditioned media (CM) was harvested and centrifuged at 300×g for 10 min to pellet debris and cells. The supernatant was transferred to a fresh tube, and EVs were isolated using an Exoquick EV Isolation Kit (SBI System Biosciences, CA, United States) according to the manufacturer’s instructions as described previously (Chugh et al., 2013; Umezu et al., 2013; Li et al., 2016; Raoof et al., 2018; Tara et al., 2018; Junling et al., 2019; Li et al., 2019; Ling et al., 2019), and the samples were stored at −80°C for use.

### Electron Microscopic Analysis of EVs

A drop of EV suspension (about 10 µL) was fixed on a formvar-coated copper grid for 2 min, washed briefly in ultrapure water, negatively stained with 1% uranyl acetate, and observed by transmission electron microscopy (TEM; JEM-2000EX; Jeol, Tokyo, Japan) at an acceleration voltage of 80 kV.

### Nanoparticle Trafficking Analysis

The size distribution of EVs was analyzed by tracking particles and sized automatically based on Brownian motion and the diffusion coefficient using Zetasizer (Malvern Panalytical, Malvern, United Kingdom) at 25°C.

### BCA Protein Assay, SDS-PAGE, and Western Blot Analyses

Total protein content was assayed using the Pierce BCA Protein Assay Kit (ThermoScientific, Waltham, MA) according to the manufacturer’s instructions. The proteins were measured using a FluorChem M Fluorescent Imaging System (ProteinSimple, Santa Clara, CA), separated by SDS-PAGE (10%), and transferred to a polyvinylidene difluoride membrane (Millipore, Billerica, MA). We used three positive markers (CD9, CD63, and TSG101) for Western blots. After blocking with 5% skim milk for 2 h, the membranes were incubated overnight at 4°C with specific antibodies against CD9, CD63 (1:1,000; Sangon Biotech, China), and TSG101 (1:1,000; Zen Biotech, China). We applied horseradish peroxidase–conjugated goat anti-rabbit IgG (H + L; 1:50,000; Jackson ImmunoResearch, West Grove, PA) as a secondary antibody for 1 h at room temperature.

### Total RNA Extraction, RNA-Seq Library Preparation, and Sequencing

We extracted total RNA from EV suspension samples using Trizol reagent (Invitrogen, Carlsbad, CA) according to the manufacturer’s instruction. RNA quantity and quality were assessed using an RNA 6000 Nano Lab-Chip Kit and Agilent 2,100 Bioanalyzer (Agilent Technologies, Inc., Santa Clara, CA) with RNA integrity number >7.0. A total amount of 3 μg total RNA per sample was used as input material for the small RNA library. Sequencing libraries were generated using NEBNext^®^ Multiplex Small RNA Library Prep Set for Illumina^®^ (NEB, United States). After cluster generation, the library preparations were sequenced on an Illumina Hiseq 2,500/2000 platform and 50 bp single-end reads were generated at the Novogene Bioinformatics Institute (Beijing, China). A total amount of 2 μg RNA per sample was used as input material for the Library preparation for lncRNA sequencing. The libraries were sequenced on an Illumina Hiseq 2,500 platform, and 125 bp paired-end reads were generated. The circRNA in the whole transcriptome project was analyzed in lncRNA sequencing, and no separate library was built.

### qRT-PCR

RNA was extracted from EVs using Trizol reagent, and RNA concentration was detected by a spectrophotometer (Nanodrop 2000; Thermo Fisher). Total RNA (1 µg) was reverse-transcribed into cDNA using the PrimeScript™ RT reagent Kit with gDNA Eraser (Takara). U6 was used as control. The 2-∆∆CT method was applied to determine relative miRNA expression levels.

### Sequence Data Analysis

For small RNA sequencing, the workflow is shown in additional Figure 1A. Raw reads in fastq format were filtered through custom perl and python scripts at first. Clean reads were obtained by removing reads with poly-N, 5′ adapter contaminants, poly A or T or G or C, those without 3’ adapter or the insert tag, and low-quality reads from raw data. Q20, Q30, and GC (Q20 and Q30 are Phred scores, which represent sequencing quality, and GC represents the percentage of bases G and C in the sequencing) content of the clean data were calculated at the same time. High-quality data were used for subsequent analyses. The small RNA tags were mapped to reference sequence by Bowtie (Langmead et al., 2009) without mismatch to analyze their expression and distribution on the reference. Mapped small RNA tags were used for searching known miRNA. Mirbase20.0 was used as reference, and modified software mirdeep2 (Friedlander et al., 2012) and srna-tools-cli (http://srna-tools.cmp.uea.ac.uk/) were used to obtain the potential miRNA and draw secondary structures. The characteristics of the hairpin structure of miRNA precursor can be used to predict novel miRNA. The available software miREvo (Wen et al., 2012) and mirdeep2 (Friedlander et al., 2012) were collaboratively used to predict novel miRNA by analyzing the secondary structure, the Dicer cleavage site, and the minimum free energy of the small RNA tags unannotated in the former steps.

**FIGURE 1:**
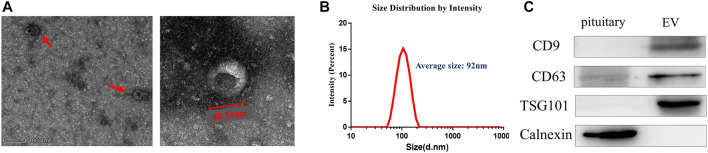
Isolation and identification of EVs from anterior pituitary of Duroc swine. **(A)** Transmission electron microscopy analysis. Wide-field **(left)** and close-up **(right).** **(B)** Size distribution analysis of EVs. **(C)** EVs confirmed by Western blot with three positive markers CD9, CD63, and TSG101.

For lncRNA sequencing, the workflow is shown in additional Figure 1B. Raw reads in fastq format were firstly processed through in-house perl scripts. Then, we obtained clean reads by removing low-quality reads and those containing adapters and poly-N from the raw data. At the same time, Q20, Q30, and GC content of the clean data were calculated. Index of the reference genome was built using bowtie2 v2.2.8 and paired-end clean reads were aligned to the reference genome using HISAT2 v2.0.4 (Langmead and Salzberg, 2012). The mapped reads of each sample were assembled by StringTie (v1.3.3) in a reference-based approach (Pertea et al., 2016). After evaluating the quality of original data produced, we set up a series of strict screening conditions according to its structural and functional characteristics based on the results of transcriptome splicing. Through the five steps screening of exon number, transcript length, known transcript annotations, transcript expression, and coding potential. The screened lncRNAs were regarded as the final candidate lncRNA set for subsequent analysis. Then, we use three types of coding potential analysis software, CNCI (Sun et al., 2013), CPC2 (Kang et al., 2017), and Pfam-scan (Punta et al., 2012), to distinguish lncRNA from mRNA. The intersecting results of each software were defined, and those that were determined to be noncoding were designated as candidate lncRNA. We used Cufflink (v2.1.1) to calculate fragments per kilobase million (FPKM) for both lncRNA and coding genes (Trapnell et al., 2010). The transcript expression levels (FPKM value) were expressed as fragments per kilobase of transcript per million mapped reads values. For circRNA sequencing, the workflow is also shown in additional Figure 1B. Quality control was carried out with the same procedures at first. Reference genome and gene model annotation files were downloaded from the genome website (NCBI Datasets) directly. Index of the reference genome was built using bowtie2 v2.2.8, and paired-end clean reads were aligned to the reference genome using Bowtie (Langmead et al., 2009). The circRNAs were detected and identified using find_circ (Memczak et al., 2013) and CIRI2 (Gao et al., 2018). Circos software was used to construct the circos figure, and the raw counts were normalized using TPM (Zhou and Zhang, 2010). We used KOBAS (Mao et al., 2005) software to test the statistical enrichment of the target gene candidates in Kyoto Encyclopedia of Genes and Genomes (KEGG) pathways. On the other hand, using miRanda, we performed ceRNA analysis, screened miRNAs and selected mRNAs, lncRNAs, and circRNAs that potentially target the miRNA and have negative expression correlations. Cytoscape software was used to construct the lncRNA-miRNA-gene and circRNA-miRNA-gene networks.

## Results

### Isolation and Identification of EVs From Anterior Pituitary of Duroc Swine

EVs were isolated from Duroc swine anterior pituitary (additional Figure 2). We detected the purified vesicles using transmission electron microscopy which showed that their size and cup-shaped morphology (Figure 1A) are typical characteristics of EVs. Then, we used Zetasizer to analyze their size distribution and found that the vesicles’ average size was about 92 nm (Figure 1B). EVs were further confirmed by Western blot with positive common surface markers CD9, CD63, and TSG101 (Figure 1C).

**FIGURE 2:**
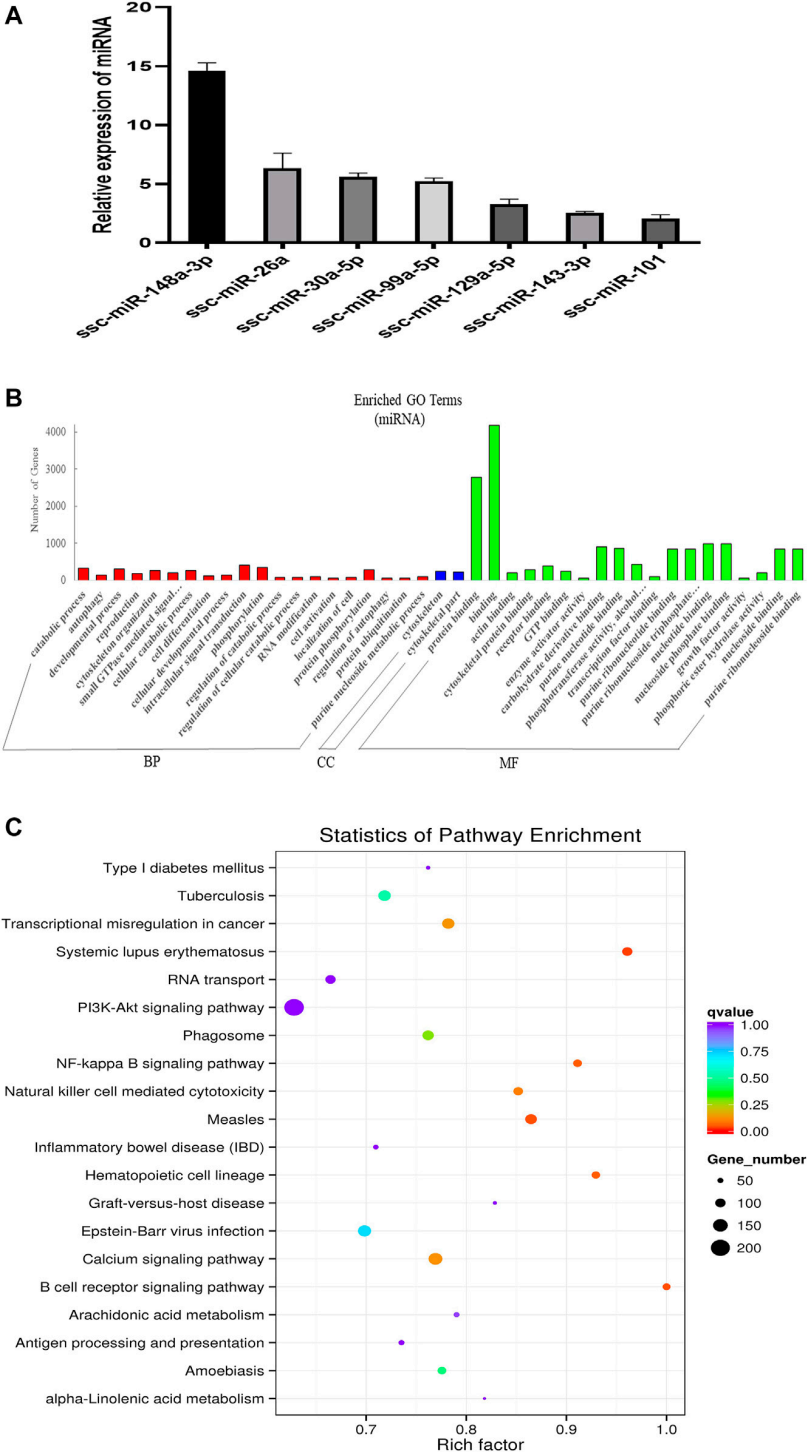
Overview and analysis of small RNA deep sequencing data in EVs. **(A)** Identification of candidate miRNAs. **(B)** Gene ontology (GO) annotation analysis. **(C)** Kyoto Encyclopedia of Genes and Genomes (KEGG) pathway analysis enrichment analysis of miRNA’s target genes. BP, biological process; CC, cellular component; MF, molecular function.

### Overview and analysis of small RNA deep sequencing data in EVs

In order to explore the ncRNA expression profiles of the EVs, we used RNA-seq analyses to characterize the ncRNA from normal anterior of three 60-day-old Duroc swine. We obtained 12778982 (EV_1), 15668033 (EV_2), and 15353011 (EV_3) clean reads that were screened from small RNA (sRNA) for subsequent analysis after quality evaluation (additional file 1: Supplementary Table S1). Meanwhile, the length distribution of the obtained total sRNA fragments were analyzed (additional Figure 3). In general, sRNAs ranged from 18 to 35 nt in length and the majority of the miRNA reads were about 22 nt. A total of 416 miRNAs were obtained from samples, 343 of which are known miRNAs and 73 are newly predicted miRNAs (additional file 2: Supplementary Table S2). Of these known miRNAs, 61 miRNAs were highly expressed (1,000 < average signals ≤ 10,000), and, in particular, 46 miRNAs were extremely highly expressed in EVs (average signals ≥10,000). We randomly selected a few candidate highly expressed miRNAs, and their relative expression levels were consistent with the sequencing results (Figure 2A). To further characterize the regulatory roles of miRNAs in the anterior pituitary EVs, miRNA target prediction, Gene Ontology (GO), and Kyoto Encyclopedia of Genes and Genomes (KEGG) annotation analyses were performed. A total of 25,516 target genes for the 416 miRNAs were predicted. Our GO annotation indicated that these predicted target genes were significantly enriched in intracellular signal transduction, phosphorylation, catabolic process, developmental process, the component of cytoskeletal part, binding, protein binding, and nucleotide-binding (Figure 2B). The KEGG pathway analysis results revealed that the genes were associated with several pathways, including NF-kappa B signaling pathway, Calcium signaling pathway and B cell receptor signaling pathway (Figure 2C). These findings suggest that miRNA in anterior pituitary EVs may be involved in regulating intracellular signal transduction and immune metabolism.

**FIGURE 3:**
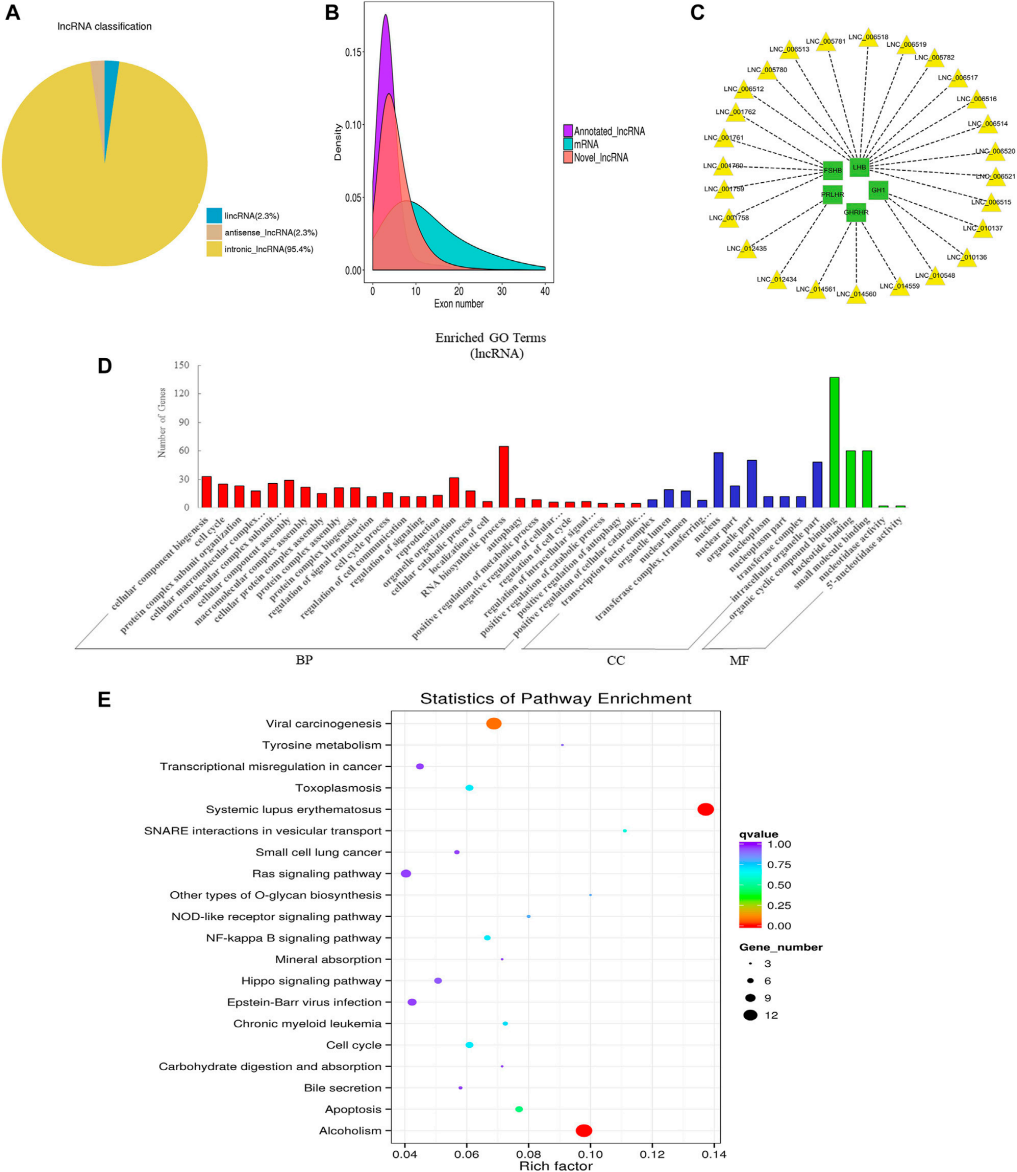
Overview and bioinformatics analysis of lncRNA deep sequencing data in EVs. **(A)** LncRNA type distribution map. **(B)** Number density map of LncRNA and mRNA exons. **(C)** lncRNA-mRNA co-expression (green and yellow represent genes and lncRNAs respectively). **(D)** GO annotation analysis. **(E)** KEGG pathway analysis enrichment analysis. BP, biological process; CC, cellular component; MF, molecular function.

### Overview and bioinformatics analysis of lncRNA deep sequencing data in EVs

LncRNA is a class of RNA molecules with transcript lengths over 200 nt and does not encode proteins. We set the filter criteria according to its characteristics and counted the number of transcripts screened per step (additional Figure 4A). For lncRNA prediction, CPC and CNCI were used for potential coding ability detection, and PFAM, a protein database, was used for protein annotation information analysis and potential coding ability detection (additional Figure 4B). Resultantly, 15,545 novel lncRNAs and 687 annotated lncRNAs (additional file 3: Supplementary Table S3) were identified respectively. We classified different types of lncRNA (lincRNA, anti-sense_lncRNA, and intronic_lncRNA), and results showed that the percentage of intronic_lncRNA was the highest (Figure 3A). The structure and sequence conservation of lncRNAs and mRNAs were also compared and analyzed. We found that lncRNAs were shorter in length in the transcript (additional Figure 4C) and their genes tended to contain fewer exons (Figure 3B). Most of the mRNAs had longer open reading frames than lncRNAs (additional Figure 4D). The transcript expression levels of lncRNAs were higher than that of mRNAs (additional Figure 4E), and we also got the same perception by comparing the FPKM of EVs from the different samples (additional Figure 4F). We investigated the possible functions of the lncRNAs by searching for protein-coding genes 100 kb upstream and downstream of all identified lncRNAs to predict the potential *cis*-regulatory targets of lncRNAs (Bao et al., 2018). A total of 16,439 protein-coding genes were predicted for 9,524 lncRNAs. A number of lncRNAs were found to co-express with pituitary-specific genes including growth hormone 1 (*GH1*), growth hormone-releasing hormone receptor (*GHRHR*), prolactin-releasing hormone receptor (*PRLHR*), follicle-stimulating hormone subunit beta (*FSHB*), and luteinizing hormone subunit beta (*LHB*) (Figure 3C). Some other lncRNAs co-expressed with genes involved in EVs’ marker protein, protein transport, and docking such as *CD63*, *CD81*, *TSG101*, *Rab27A*, *Rab27B*, and *UBL3*. GO annotation indicated that the predicted target genes of lncRNAs were significantly enriched in cellular component biogenesis, organelle organization, RNA biosynthetic process, the cellular component of nucleus and organelle part, organic cyclic compound binding, nucleotide binding, and small molecule binding (Figure 3D). KEGG pathway analysis revealed that these genes were associated with Systemic lupus erythematosus, alcoholism, apoptosis, cell cycle, and NF-kappa B signaling pathway (Figure 3E). These data indicated that lncRNAs in EVs of the anterior pituitary could participate in the immune regulation and growth process of organisms.

**FIGURE 4:**
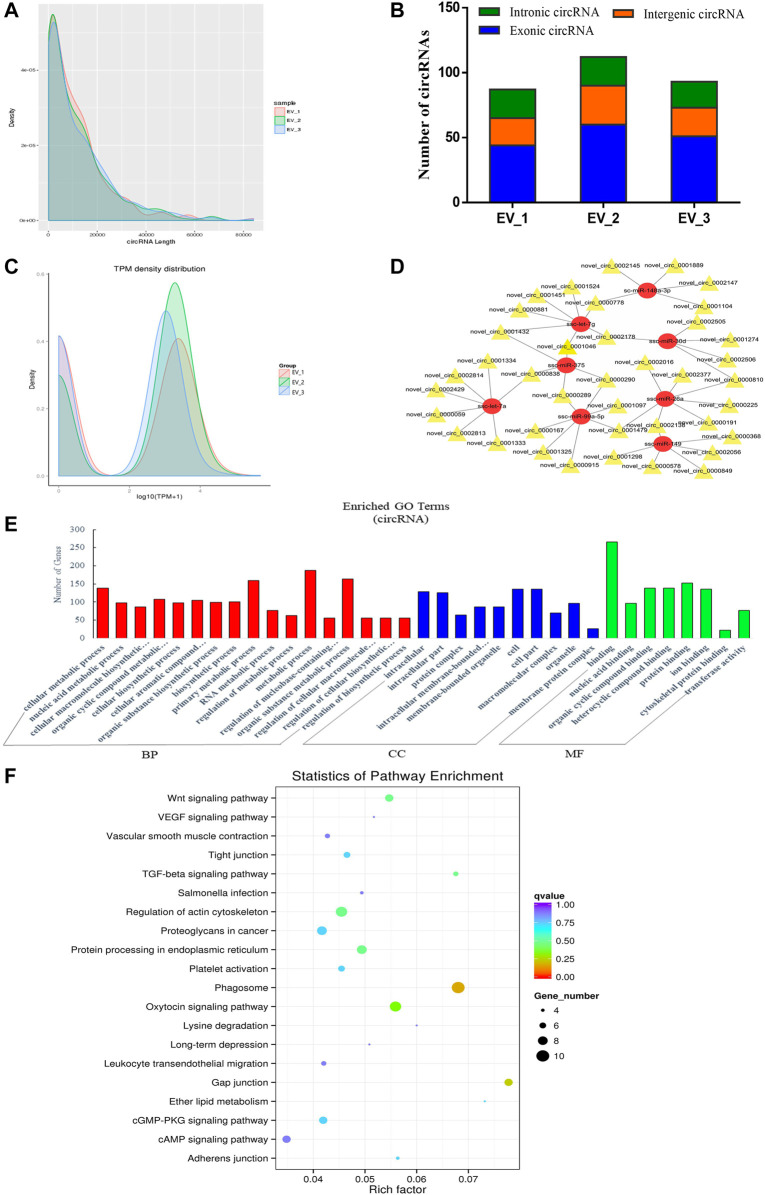
Overview and bioinformatics analysis of circRNA deep sequencing data in EVs. **(A)** The length distribution of circRNAs for all samples. **(B)** The source of circRNAs for all samples, showing the numbers of exonic, intronic and intergenic circRNAs of each sample. **(C)** TPM density map, showing consistency between samples. **(D)** The network of circRNA-miRNA co-expression (red and yellow represent miRNA and circRNA respectively). **(E)** GO annotation analysis **(F)** KEGG pathway analysis enrichment analysis. BP, biological process; CC, cellular component; MF, molecular function.

### Overview and bioinformatics analysis of circRNA deep sequencing data in EVs

After evaluating the data output quality, we obtained 495 novel circRNAs (additional file 4: Supplementary Table S4) and then counted the length distribution and the source of the circRNAs for all samples (Figure 4A). It showed that the length of the circRNAs is mostly scattered in a range of less than 10000 nt and the sources of the circRNAs mostly from the intergenic area compared with the exon and intron area (Figure 4B). The expression levels of all circRNAs were statistically analyzed and normalized by TPM (Figure 4C). TPM density distribution allows overall inspection of gene expression patterns in samples, and the results showed large overlap areas which meant a consistency between samples (Zhou et al., 2010). We then constructed a circRNA-miRNA co-expression network based on the RNA-seq results. CircRNA could inhibit the function of miRNA by combining it with miRNA (Hansen et al., 2013). Therefore, the analysis of miRNA binding sites on the identified circRNAs helps to further study the function of circRNAs. Then, we used miRanda to predict the miRNA binding sites of cleaved circRNAs and eventually focused on those circRNAs that combined with highly expressed miRNAs in the pituitary and EVs from the anterior pituitary. A network map was constructed containing 39 circRNAs, 8 miRNAs, and 49 relationships (Figure 4D). In order to explore the potential functions of the circRNAs in EVs from the anterior pituitary, we performed GO and KEGG pathway enrichment analysis. The results showed that the enriched GO terms were mainly associated with metabolic process, cellular biosynthetic process, binding, and transferase activity (Figure 4E) and the KEGG pathways were mainly enriched in Phagosome, gap junction, the Wnt signaling pathway, regulation of actin cytoskeleton, and protein processing in endoplasmic reticulum (Figure 4F). These findings indicated that circRNAs in EVs of the anterior pituitary could regulate the cellular metabolic and biosynthetic process.

### Analysis of crosstalk in lncRNA-miRNA-mRNA relationship in EVs

**FIGURE 5:**
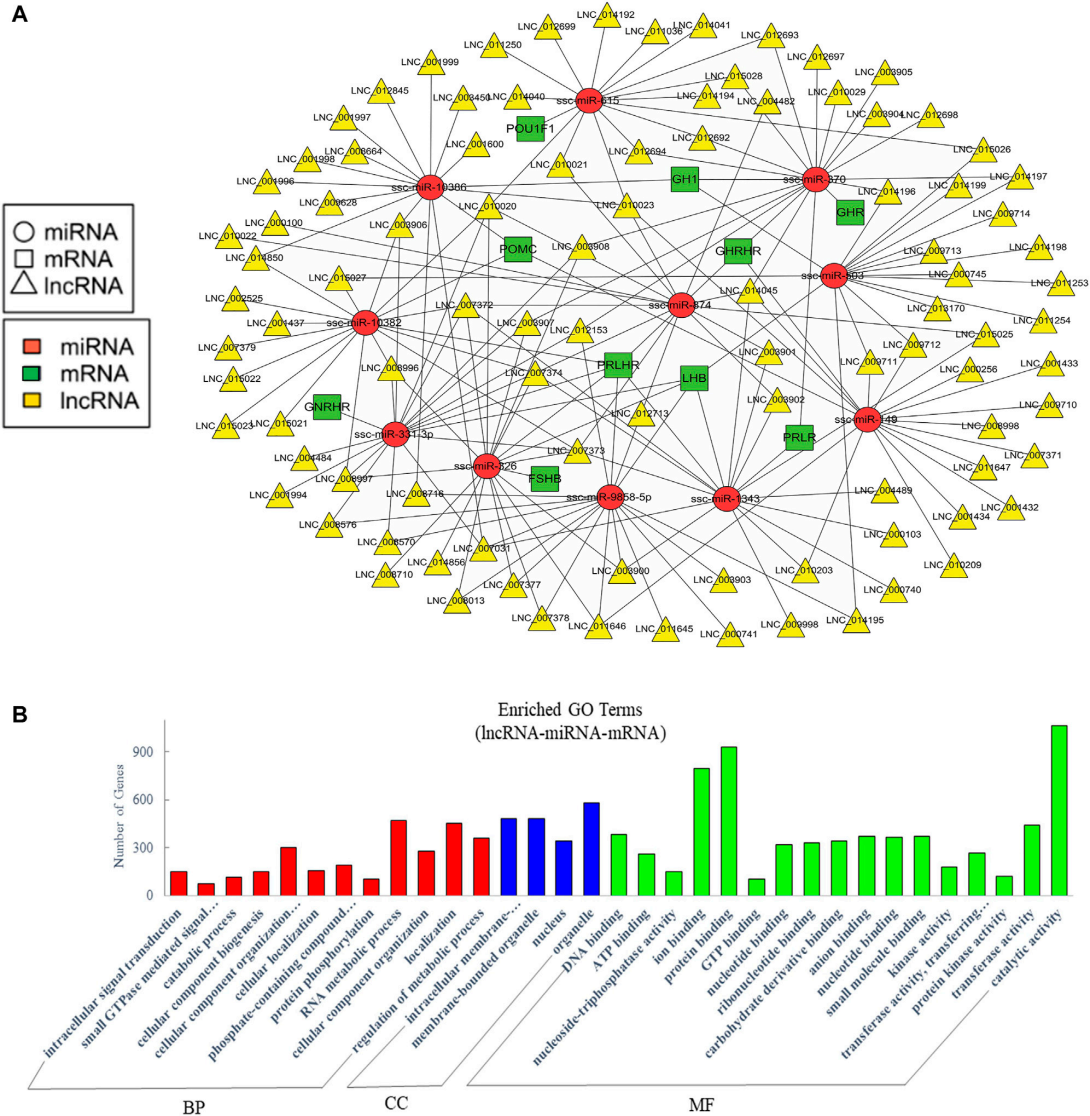
Analysis of crosstalk in lncRNA-miRNA-mRNA relationship in EVs. **(A)** The ceRNA network of lncRNA, miRNA and pituitary-specific genes. **(B)** GO annotation analysis. GO analysis show significantly enriched terms (*p* < 0.05) in the categories of biological process, cellular components, and molecular function. BP, biological process; CC, cellular components; MF, molecular function.

Recent studies suggested that lncRNAs could function as endogenous miRNA sponges to prevent miRNA from binding to reduce the regulatory effect of miRNAs on their target mRNA (Cai and Cullen, 2007; Wang et al., 2010; Tay et al., 2014). To further analyze the crosstalk between lncRNA, miRNA, and mRNA, we predicted their interaction and further focused on the competitive endogenous RNAs (ceRNAs) relative with pituitary function. A network was drawn with 97 lncRNAs that could sponge 11 miRNAs to regulate 10 pituitary-specific genes including *GH1*, *GHRHR*, *PRLHR*, *FSHB*, *LHB*, proopiomelanocortin (*POMC*), growth hormone receptor (*GHR*), prolactin receptor (*PRLR*), gonadotropin-releasing hormone receptor (*GNRHR*), and POU class 1 homeobox 1 (*POU1F1*) (Figure 5A). We also performed GO enrichment analysis, which revealed 273 significantly enriched terms in the categories of biological process, molecular function, and cellular components, and we showed a part of terms with lots of gene numbers (Figure 5B). Its annotation indicated that they participated in intracellular signal transduction, cellular component organization or biogenesis, RNA metabolic process, localization, regulation of the metabolic process, binding, and regulation of catalytic activity which suggested that they were involved in the body’s basic biological regulation.

### Analysis of crosstalk in circRNA-miRNA-mRNA relationship in EVs

**FIGURE 6:**
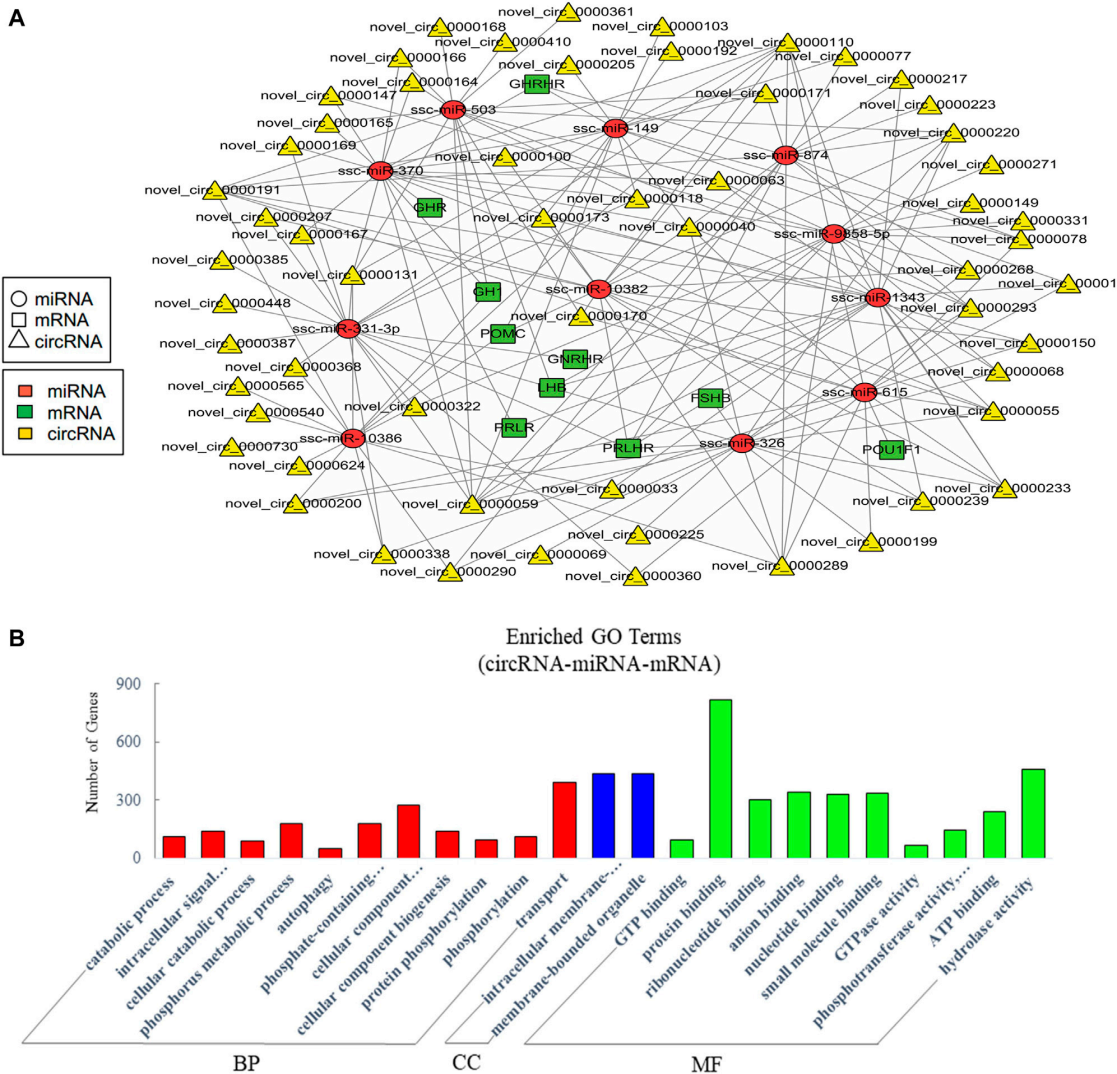
Analysis of crosstalk in circRNA-miRNA-mRNA relationship in EVs. **(A)** The ceRNA network of circRNA, miRNA, and pituitary-specific genes. **(B)** GO annotation analysis, showing significantly enriched terms (*p* < 0.05) in the categories of biological process, cellular components, and molecular function. BP = biological process; CC = cellular components; MF = molecular function.

The current studies have proved that circRNAs could act as ceRNAs to compete for miRNA-binding sites to affect the function of miRNAs (Hansen et al., 2013; Thomas and Saetrom, 2014). Therefore, the analysis of interactions between miRNAs and circRNAs is helpful for further study. Similarly, we mainly concerned the ceRNAs relative to pituitary function in the constructed potential circRNA–miRNA–mRNA associations. The resultant network was comprised of 188 edges among 11 miRNAs, 58 circRNAs, and 10 pituitary-specific genes including *GH1*, *POMC*, *GHR*, *GHRHR*, *PRLR*, *LHB*, *PRLHR*, *FSHB*, *GNRHR*, and *POU1F1* (Figure 6A). For the potential functions of the associated ncRNAs in EVs from the anterior pituitary, we conducted GO enrichment analysis which revealed 265 significantly enriched terms. Some terms enriched a high number of genes (Figure 6B). Our GO annotation indicated that they were involved in intracellular signal transduction, cellular component organization or biogenesis, transport, protein binding, hydrolase activity, and phosphotransferase activity. These findings suggested that the network in circRNA-miRNA-mRNA relationship in EVs played an important role in the process of biosynthetic and information transmission.

## Discussion

EVs contain plentiful cargoes including proteins, lipids, and nucleic acids which are specifically sorted and packaged, and contents packed are cell type-specific (Hessvik and Llorente, 2018). More and more evidence indicated that EVs can transfer important cargoes such as miRNA, mRNA, and proteins from cell to cell *via* membrane vesicle delivery, thereby being a new approach of intracellular or organ-to organ communication (Théry et al., 2002; Mathivanan et al., 2010; Tan et al., 2013; Javeed and Mukhopadhyay, 2017). Studies have reported that EVs can mediate the transmission of information between endothelial cells, smooth muscle cells, cardiomyocytes, stem cells, and fibroblasts (Hergenreider et al., 2012; Bang et al., 2014; Wang et al., 2014). Hepatocyte-derived EVs could act as potential biomarkers of liver disease and promote cell proliferation and liver regeneration (Bala et al., 2012; Nojima et al., 2015). EVs secreted by skeletal muscle contain proteins and miRNAs that can be transferred to adjacent muscle cells (Pedersen and Febbraio, 2012). EVs from the adipose tissue could mediate activation of macrophage-induced insulin resistance and are regarded as the main immune regulator secreted by insulin resistance factors (Deng et al., 2009; Kranendonk et al., 2014). As an important endocrine gland, whether the pituitary gland produces EVs and its cargos remains unclear up to date.

Firstly, considering that the pig pituitary is small and difficult to obtain, we used the Exoquick Isolation Kit to isolate the EVs, then identified them using transmission electron microscopy and western blot detection of CD9, CD63, TSG101, and Calnexin, followed by RNA extraction and sequencing. The Venn diagrams of miRNAs, lncRNAs, and circRNAs were drawn through analysis to show the distributions of numbers among three samples (additional Figure 5). A total of 416 miRNAs were obtained from samples, 343 of which are known miRNAs and 73 are newly predicted miRNAs. Our research group has revealed the expression of miRNAs in porcine anterior pituitary cells and found that miRNAs could regulate the hormone secretion from the anterior pituitary (Qi et al., 2015, Ye, et al., Ye et al., 2013). Interestingly, we found most of the top 20 miRNAs such as miR-7, miR-375, let-7a, let-7c, miR-26a, miR-30a, let-7g, miR-30days, miR-127, miR-151, miR-21, miR-149, miR-99a, and miR-143 in EVs are also highly expressed in the porcine pituitary (Yuan et al., 2015). Various studies also revealed several of their enrichment in metabolisms and functions. MiR-7 is abundant in the pituitary of mice (Bak et al., 2008) and pigs (Ye et al., 2015; He et al., 2018). Research showed that miR-7 might play an important role in the hypothalamic–pituitary–gonadal (HPG) axis and be involved in body growth by acting on the pituitary *GHRHR* in pigs (Zhang et al., 2013a; He et al., 2018). MiR-375 could regulate pituitary pro-opiomelanocortin (*POMC*) expression (Zhang et al., 2013b). Let-7f-5p was a highly expressed miRNA of the let-7 family in the pituitary (Wu et al., 2017). Mir-26a plays an important role in cell cycle control by modulating protein kinase C delta (Erica et al., 2013). MiR-200b could stimulate luteinizing hormone (LH) levels by targeting *ZEB1* (Hasuwa et al., 2013). KEGG and GO analysis suggest that miRNAs in EVs of the anterior pituitary could regulate intracellular signal transduction, phosphorylation, catabolism, and development.

CeRNAs regulate gene expression by competitively binding to microRNAs (Salmena et al., 2011). Recent studies have shown that the interaction of the miRNA seed region with mRNA is not unidirectional, but that the pool of mRNAs, lncRNA (Cesana et al., 2011), and circRNA (Hansen, et al., Memczak, et al.) competes for the same library of miRNA to regulate miRNA activity (Tay et al., 2011). These ceRNAs act as molecular sponges for miRNA through their miRNA binding sites to inhibit target genes of the respective miRNA family. Unlike miRNAs, the function of lncRNAs and circRNAs is poorly understood in pig pituitary.

There are a large number of studies that have identified the role of lncRNA in pituitary function. Researches have shown that the anterior pituitary lncRNA of rats plays an important role in hormone and reproduction development and regulation (Han et al., 2017). MIR205HG enabled to regulate the secretion of GH and PRL in anterior pituitary (Du et al., 2019). LncRNA C5orf66-AS1 suppressed the development and invasion of pituitary null cell adenomas (Yu et al., 2017). LncRNA RPSAP52 was verified to act as miRNA sponge to promote cell growth (D'Angelo et al., 2019). In our study, some lncRNAs could co-express with pituitary-specific genes like *GH1*, *GHRHR*, *PRLHR*, *FSHB*, and *LHB.* Some other lncRNAs could co-express with genes involved in EVs’ marker protein, protein transport, and docking such as *CD63*, *CD81*, *TSG101*, *Rab27A*, *Rab27B*, and *UBL3*. On the other hand, the signal of a two-circRNA was found to be able to predict tumor recurrence in clinically non-functioning pituitary adenoma (Guo et al., 2019). Another study reported that thousands of sheep genes could express circRNAs in the pituitary gland (Li et al., 2017a). Regarding circRNA, in this article, we determined numerous circRNAs that interact with highly expressed miRNAs both in EVs and the pituitary, participating in the biologic functions of the pituitary gland. Our results suggests that ceRNAs in EVs from the anterior pituitary may take part in the cellular metabolic and biosynthetic process and the cross-talk between mRNA, miRNA, lncRNA, and circRNA may be involved in the regulation of pituitary endocrine functions and signaling process. Since EVs are composed of complicated groups with different size and contents, methods for extraction and purification of them are still in development. The extraction kit used in this study is well accepted nowadays, though it has potential limitations and we will further verify and explore the function of obtained ncRNAs in subsequent research.

On the whole, our study is the first exploration of the expression of ncRNAs in EVs delivered by the anterior pituitary in Duroc swine model. MiRNAs, lncRNAs, and circRNAs of EVs from the anterior pituitary may act as novel regulators of pituitary development and endocrine regulation. These findings provided an insight into EVs derived from the anterior pituitary and are helpful to explore the potential functions of EV cargoes.

## Data Availability

The original contributions presented in the study are publicly available in NCBI under accession number PRJNA644768.

## Abbreviations

ACTH, adrenocorticotropin hormone; ceRNAs, competitive endogenous RNAs; circRNA, circular RNA; FSH, follicle-stimulating hormone; FSHB, follicle stimulating hormone subunit beta; GH, growth hormone; GH1, growth hormone 1; GHR, growth hormone receptor; GNRHR, gonadotropin releasing hormone receptor; GHRHR, growth hormone releasing hormone receptor; GO, Gene ontology; KEGG, Kyoto Encyclopedia of Genes and Genomes; LH, luteinizing hormone: LHB, luteinizing hormone subunit beta; lncRNA, long non-coding RNA; miRNA, microRNA; ncRNA, Non-coding RNA; POMC, proopiomelanocortin; POU1F1, POU class 1 homeobox 1; PRL, prolactin; PRLHR, prolactin releasing hormone receptor; PRLR, prolactin receptor; TSH, thyroid-stimulating hormone.
