# Supplementary figures and images for: An Exploration of Non-Coding RNAs in Extracellular Vesicles Delivered by Swine Anterior Pituitary
Source: Front Genet. 2021 Nov 29;12:772753. doi: 10.3389/fgene.2021.772753 (PMC8667663; doi:10.3389/fgene.2021.772753)

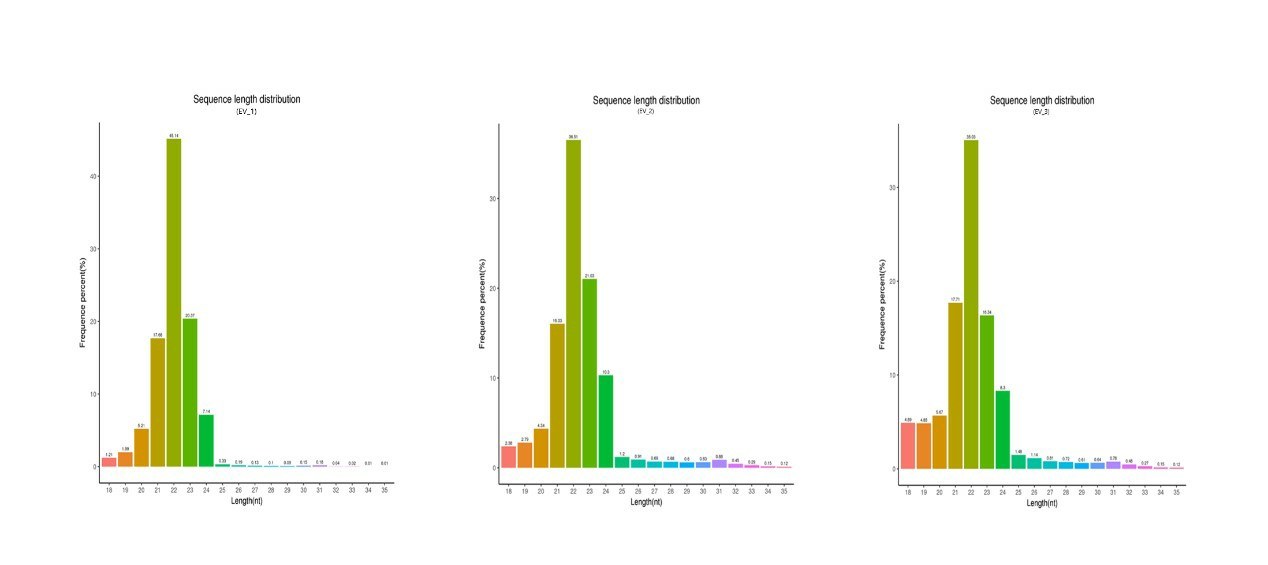

Supplement: Supplementary file 2 [file Image3.JPEG]

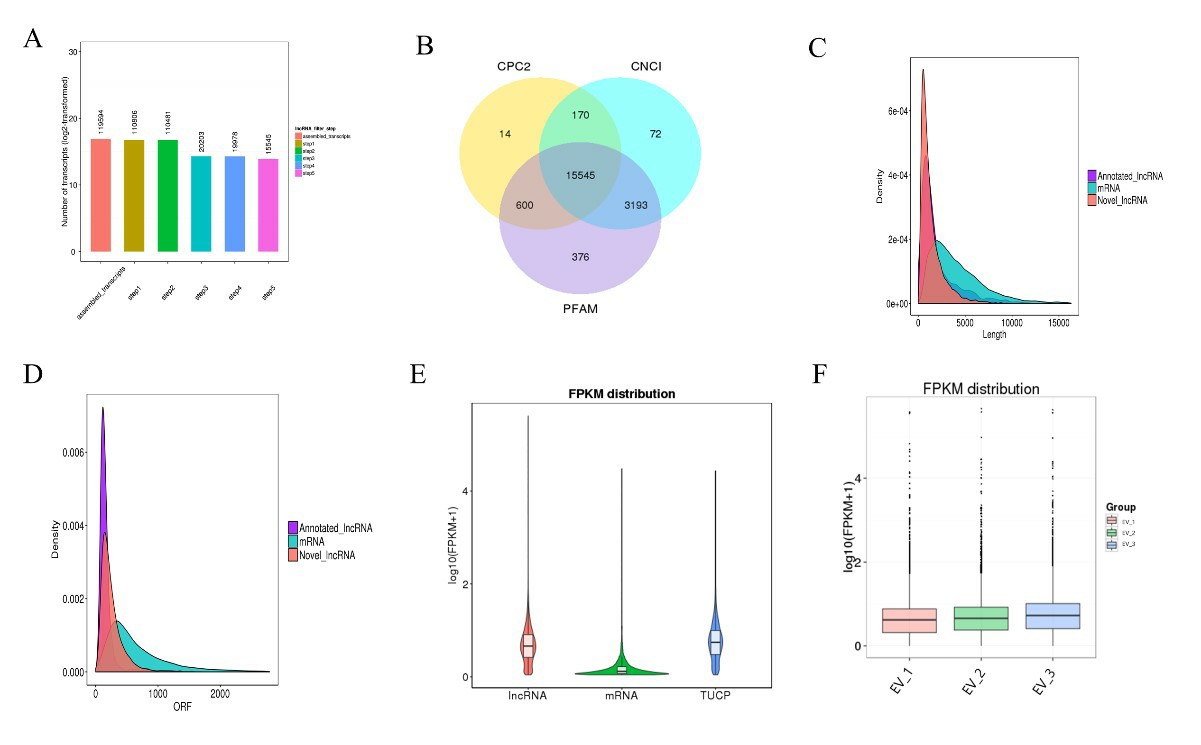

Supplement: Supplementary file 4 [file Image4.JPEG]

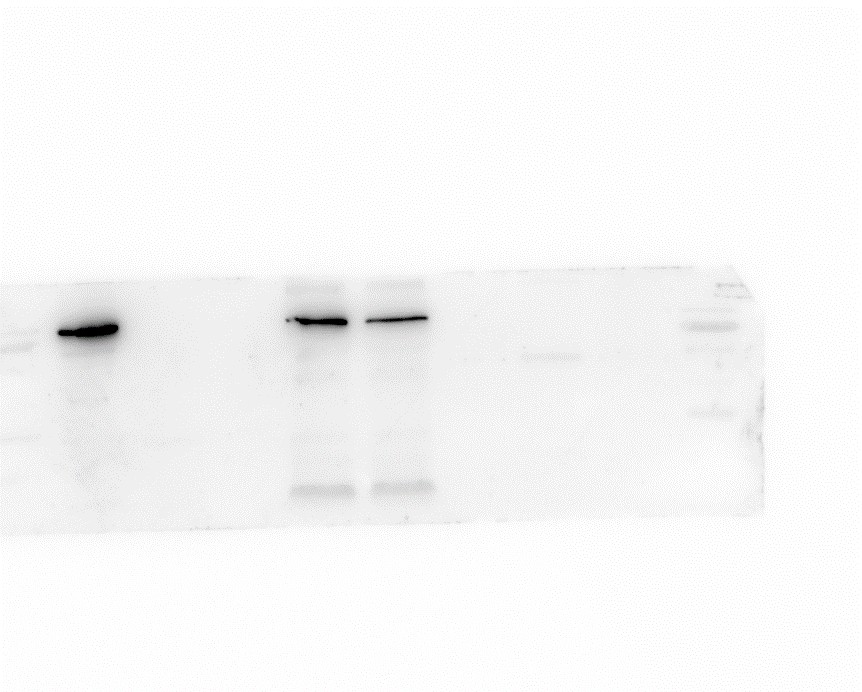

Supplement: Supplementary file 5 [file DataSheet1.ZIP › original blot/Figure 1C-Calnexin.jpg]

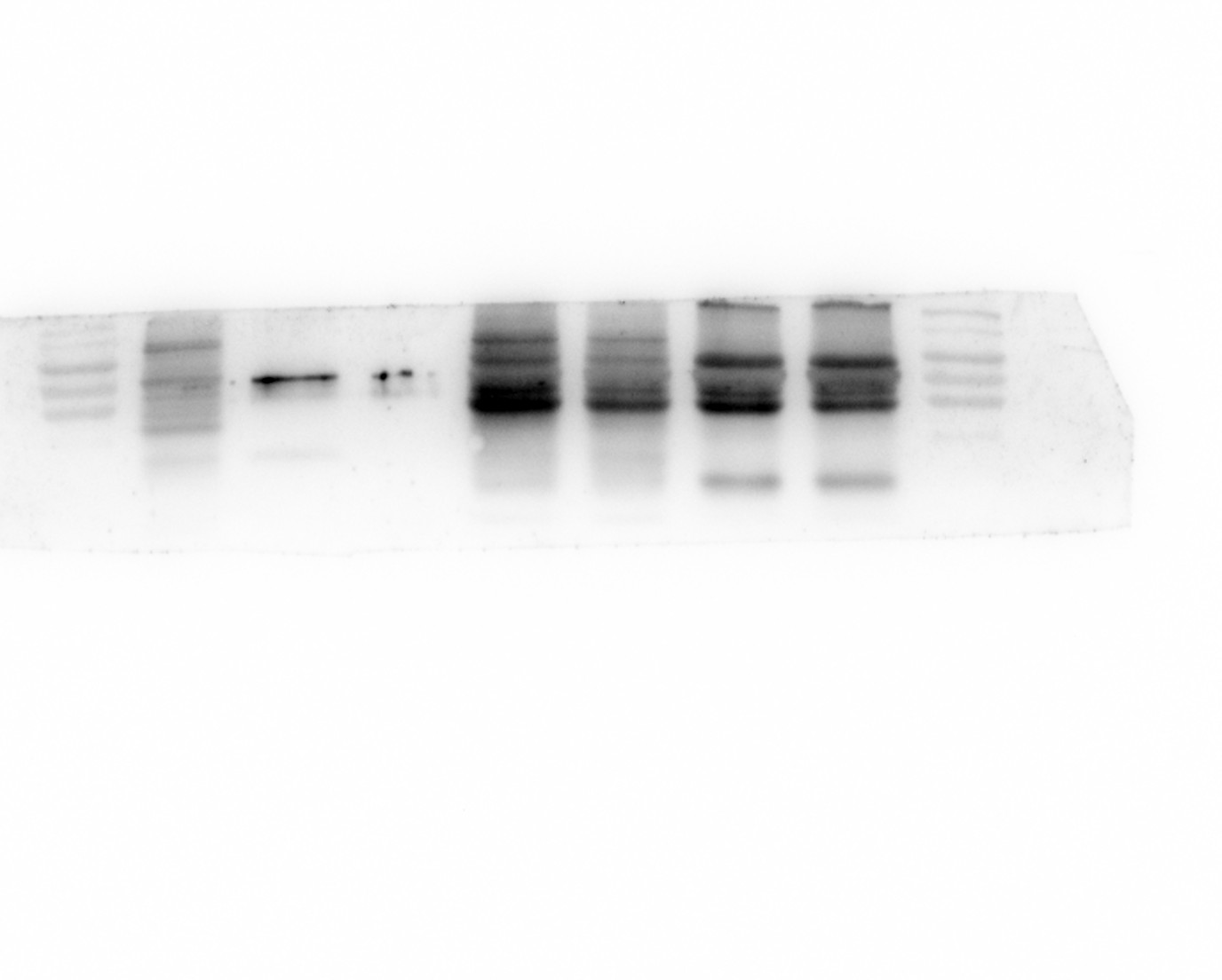

Supplement: Supplementary file 5 [file DataSheet1.ZIP › original blot/Figure 1C-CD63.jpg]

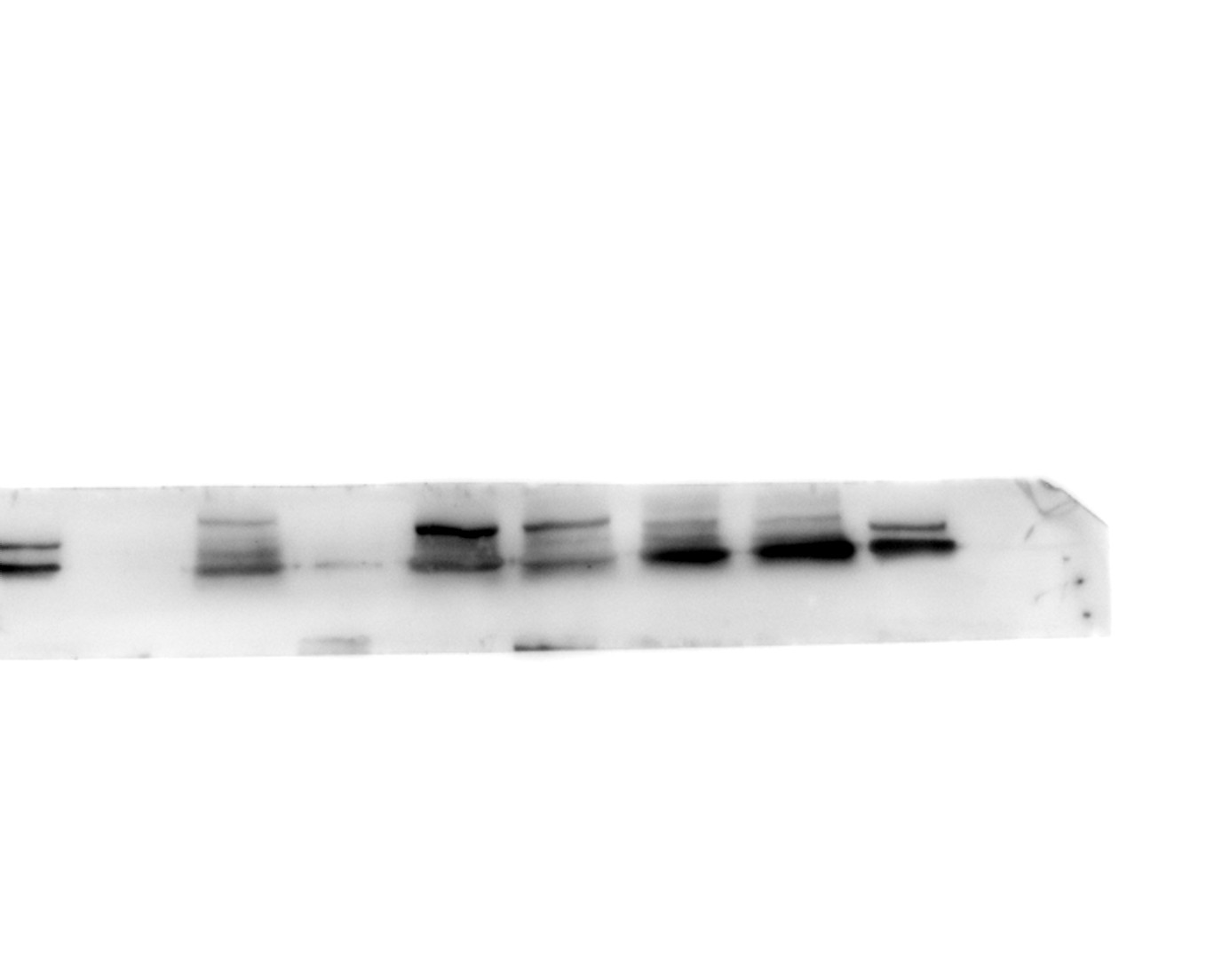

Supplement: Supplementary file 5 [file DataSheet1.ZIP › original blot/Figure 1C-CD9.jpg]

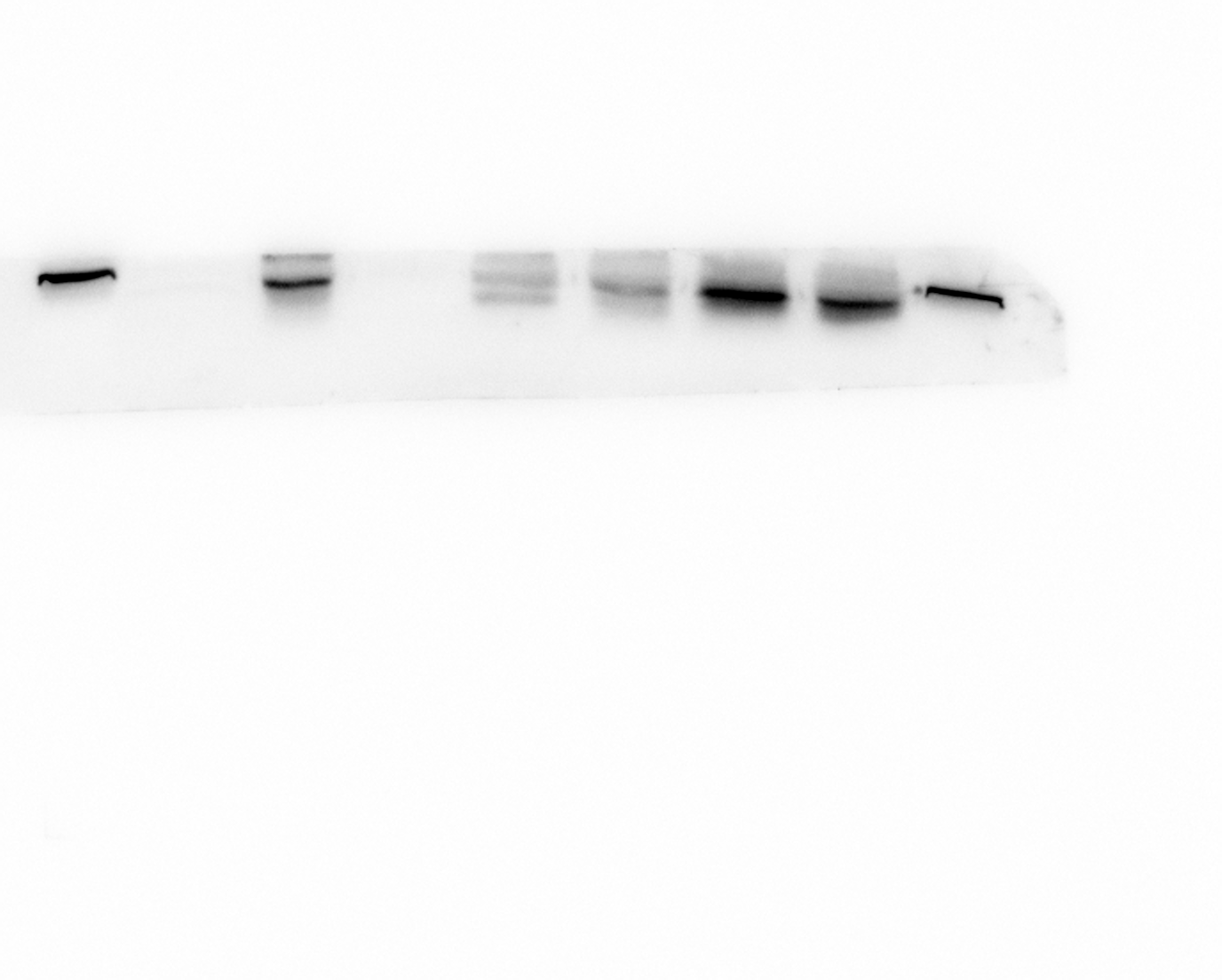

Supplement: Supplementary file 5 [file DataSheet1.ZIP › original blot/Figure 1C-TSG101.Tif]

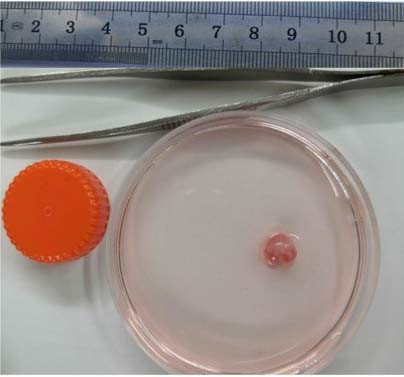

Supplement: Supplementary file 6 [file Image2.JPEG]

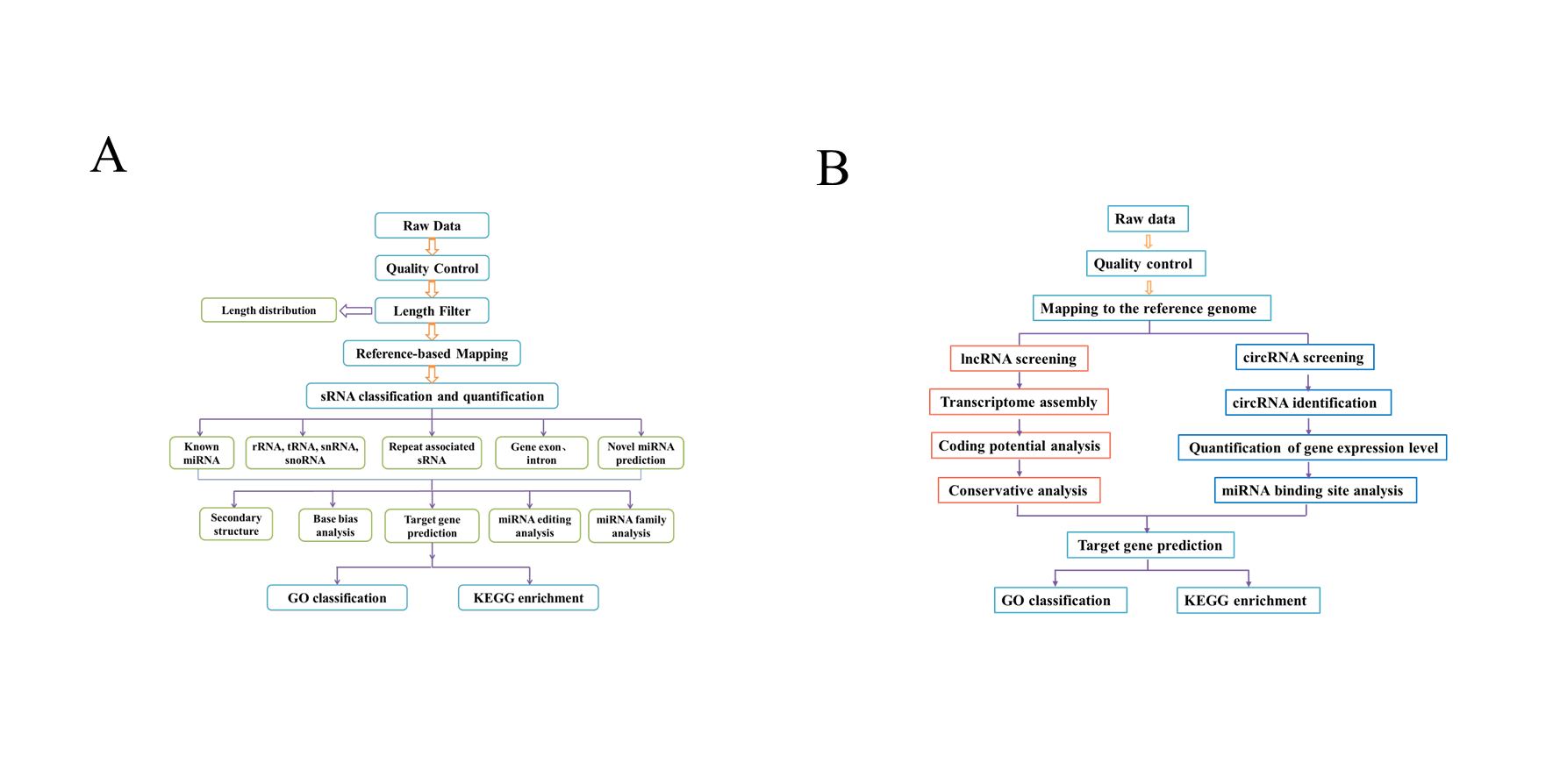

Supplement: Supplementary file 7 [file Image1.TIF]

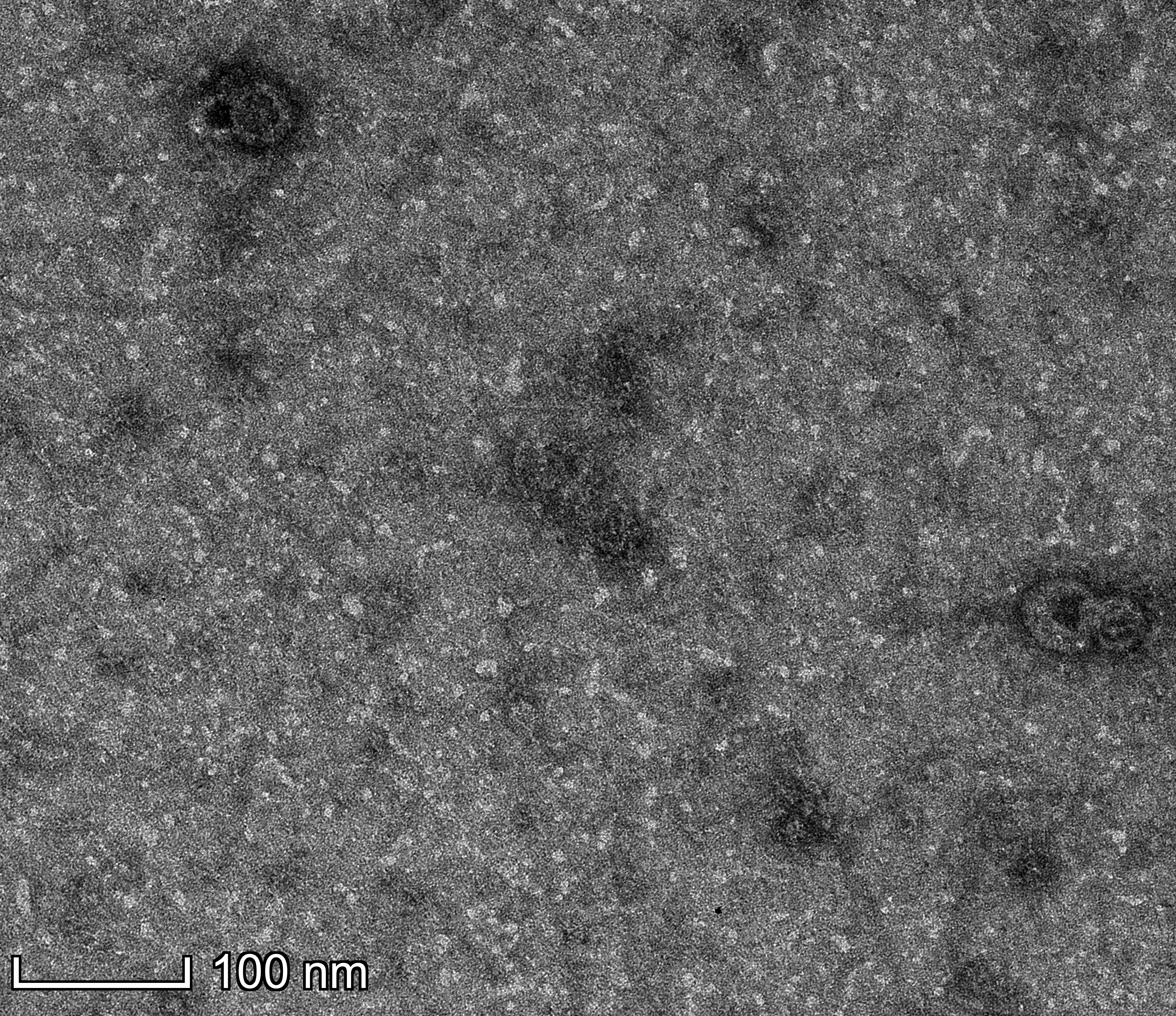

Supplement: Supplementary file 9 [file DataSheet2.ZIP › original picture of figure 1A/Picture on the left of figure 1A.tif]

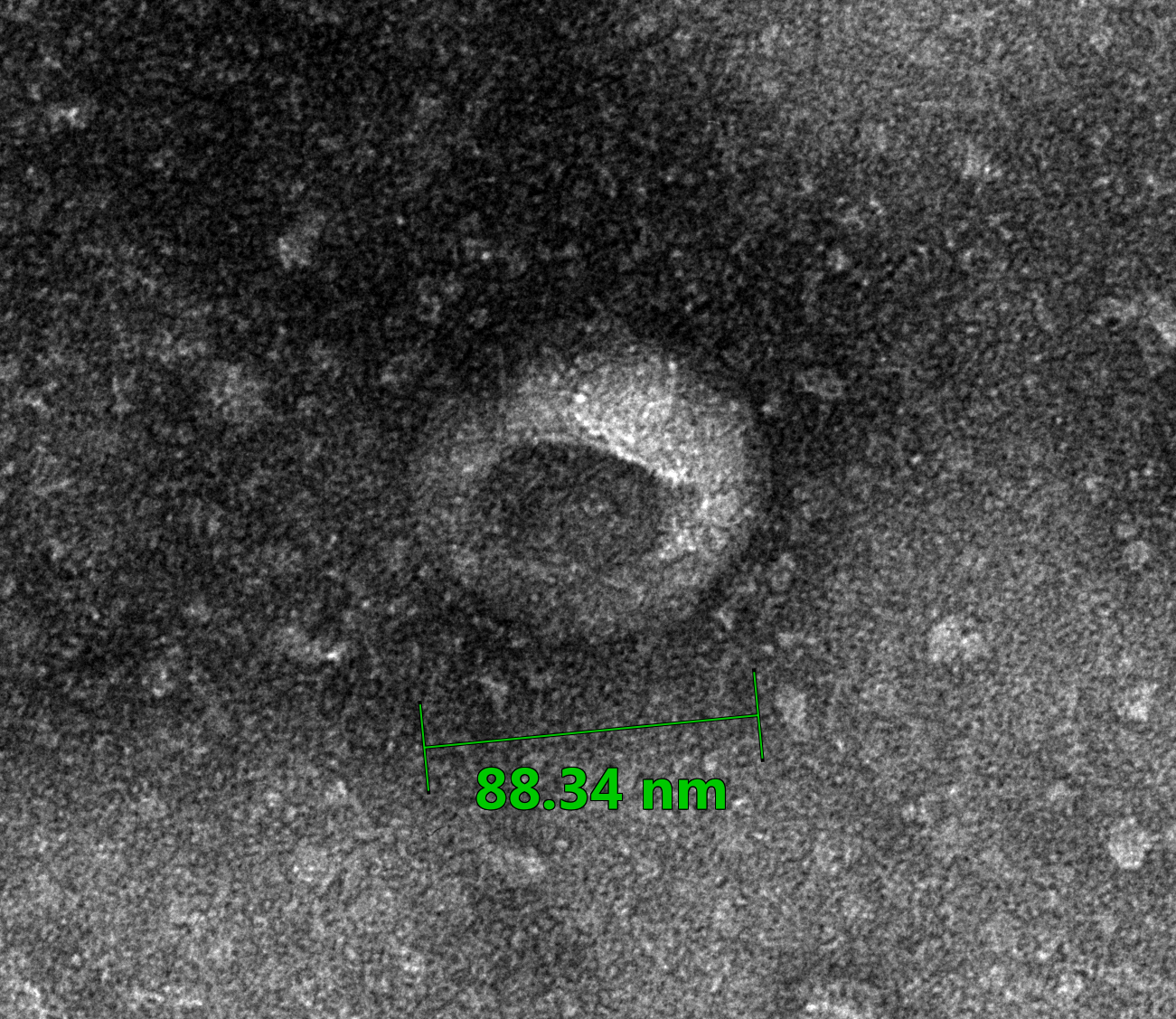

Supplement: Supplementary file 9 [file DataSheet2.ZIP › original picture of figure 1A/Picture on the right of figure 1A.tif]

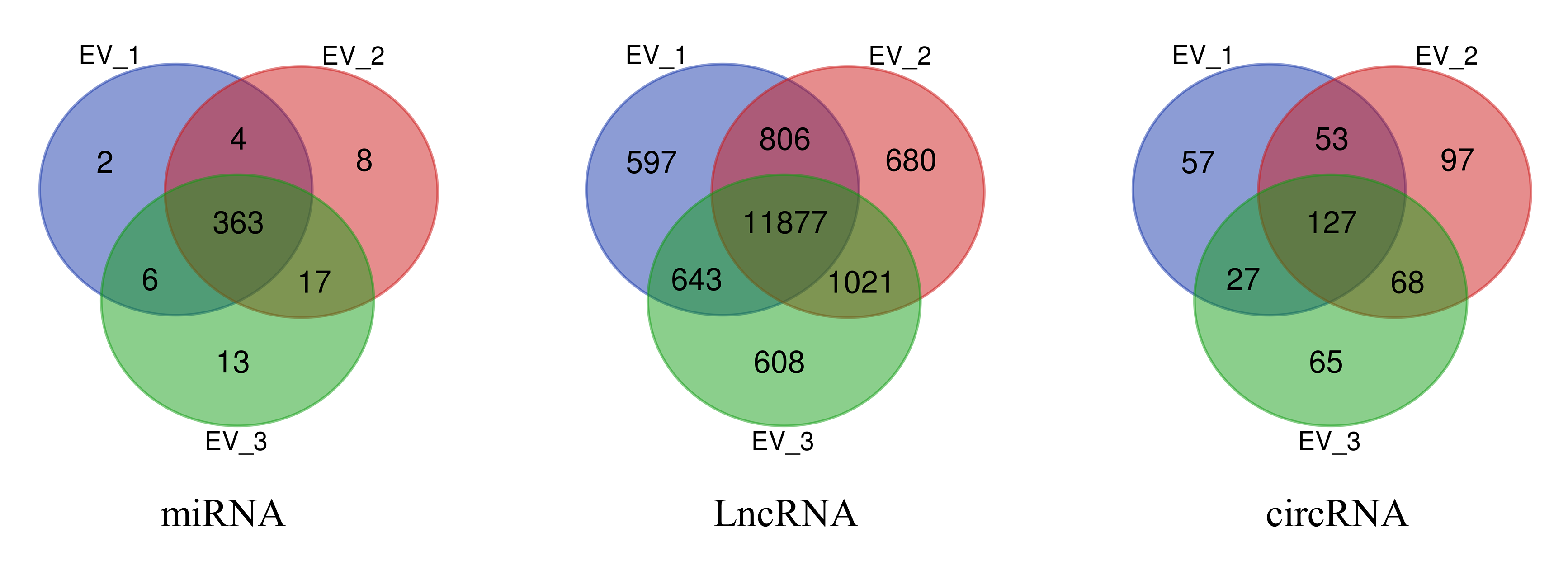

Supplement: Supplementary file 11 [file Image5.TIF]
