# Supplementary material for: An Exploration of Non-Coding RNAs in Extracellular Vesicles Delivered by Swine Anterior Pituitary
Source: Front Genet. 2021 Nov 29;12:772753. doi: 10.3389/fgene.2021.772753 (PMC8667663; doi:10.3389/fgene.2021.772753)
Supplement: Supplementary file 5 [file DataSheet1.ZIP › original blot/original western blots.docx]

**original western blots**

Figure 1-c (CD9)

**Pituitary EV**





**marker**

**marker**

Figure 1-c (CD63)





**Pituitary EV**

**marker**

**marker**

Figure 1-c (TSG101)





**marker**

**marker**

**Pituitary EV**

Figure 1-c (Calnexin)





**Pituitary EV**

**marker**

**marker**
